# From Walking to Running: 3D Humanoid Gait Generation via MPC

**DOI:** 10.3389/frobt.2022.876613

**Published:** 2022-08-16

**Authors:** Filippo M. Smaldone, Nicola Scianca, Leonardo Lanari, Giuseppe Oriolo

**Affiliations:** Dipartimento di Ingegneria Informatica, Automatica e Gestionale, Sapienza University of Rome, Rome, Italy

**Keywords:** humanoid robot, model predictive control, legged locomotion, running, variable height inverted pendulum model

## Abstract

We present a real time algorithm for humanoid 3D walking and/or running based on a Model Predictive Control (MPC) approach. The objective is to generate a stable gait that replicates a footstep plan as closely as possible, that is, a sequence of candidate footstep positions and orientations with associated timings. For each footstep, the plan also specifies an associated reference height for the Center of Mass (CoM) and whether the robot should reach the footstep by walking or running. The scheme makes use of the Variable-Height Inverted Pendulum (VH-IP) as a prediction model, generating in real time both a CoM trajectory and adapted footsteps. The VH-IP model relates the position of the CoM to that of the Zero Moment Point (ZMP); to avoid falling, the ZMP must be inside a properly defined support region (a 3D extension of the 2D support polygon) whenever the robot is in contact with the ground. The nonlinearity of the VH-IP is handled by splitting the gait generation into two consecutive stages, both requiring to solve a quadratic program. Thanks to a particular triangular structure of the VH-IP dynamics, the first stage deals with the vertical dynamics using the Ground Reaction Force (GRF) as a decision variable. Using the prediction given by the first stage, the horizontal dynamics become linear time-varying. During the flight phases, the VH-IP collapses to a free-falling mass model. The proposed formulation incorporates constraints in order to maintain physically meaningful values of the GRF, keep the ZMP in the support region during contact phases, and ensure that the adapted footsteps are kinematically realizable. Most importantly, a stability constraint is enforced on the time-varying horizontal dynamics to guarantee a bounded evolution of the CoM with respect to the ZMP. Furthermore, we show how to extend the technique in order to perform running on tilted surfaces. We also describe a simple technique that receives input high-level velocity commands and generates a footstep plan in the form required by the proposed MPC scheme. The algorithm is validated via dynamic simulations on the full-scale humanoid robot HRP-4, as well as experiments on the small-sized robot OP3.

## 1 Introduction

Humanoid robots have become more and more advanced in the last decade. Using legged locomotion, they are in principle able to perform very complex and dynamic motions, such as those needed to traverse nonlevel terrain and realize running gaits. However, the design of online walking and running pattern generation algorithms is challenging from a control standpoint, as there are several hard requirements such as the need to always maintain dynamic balance, whether the robot is walking or running.

When walking on flat ground, balance is usually guaranteed by keeping the Zero Moment Point^1^ (ZMP) within the convex hull of the contact surfaces, that is, the support polygon. To achieve real time control, a popular approach is to reduce the complexity of humanoid dynamics using the Linear Inverted Pendulum (LIP) model, obtained by assuming constant CoM height and neglecting angular momentum variations around the CoM. The linearity of the LIP model has allowed to efficiently define control schemes based on optimization, such as preview-based approaches for tracking the desired ZMP trajectory (Kajita et al., 2003), or MPC schemes that encode the balance requirement through constraints on the ZMP (Wieber, 2006).

### 1.1 Related Work

Variation of the CoM height is a key requirement for traversing nonlevel grounds. Removing the assumption of constant CoM height in the LIP leads to the Variable-Height Inverted Pendulum (VH-IP) model (Koolen et al., 2016), in which height variations have the effect of modifying the natural frequency of the pendulum. The varying natural frequency can be seen as an additional input of the model, which introduces a nonlinearity that entails higher complexity in the gait generation algorithm. Another possibility is to consider it as a time-varying parameter in the dynamic equation (Herdt et al., 2012), design the vertical motion offline, and generate the horizontal trajectories with a linear time-varying MPC. Other approaches (Hu et al., 2014; Brasseur et al., 2015) aim instead at bounding the effect of the difference between the VH-IP and LIP on the ZMP, in a way to guarantee that the latter is always inside the support polygon. It is also possible to find specific trajectories for which the vertical CoM motion has LIP-like dynamics (Englsberger et al., 2013; Zamparelli et al., 2018). This approach can be very effective but it constrains the CoM trajectories that can be generated.

Regardless of the employed model, gait generation schemes must necessarily deal with the intrinsic instability of humanoid dynamics. In the LIP, this instability is reflected in the presence of an unstable mode. This component is often called Divergent Component of Motion (DCM) (Takenaka et al., 2009a), or Capture Point (Pratt et al., 2006), and plays a major role in the stabilization of gait generation schemes. In the effort to extend this concept to the VH-IP, if one follows the same approach as for the LIP, it is hard to enforce stability on the DCM itself as it will remain coupled with the CoM dynamics (Hopkins et al., 2014). However, a truly decoupling change of coordinates can be found, (Caron et al., 2019a), but it is a time-varying change of coordinates with no available closed-form expression, as it is implicitly defined by solving a nonlinear differential equation.

A separate line of research has been directed at the generation of running motions, with early work dating back several decades (Raibert, 1986). To generate a running gait, the controller must be able to produce a variable CoM height, but it also needs to account for flight phases, in which contact with the ground is lost. As such, none of the aforementioned control schemes for 3D walking can be directly adopted to generate a running gait. Biomechanic studies on human running (Blickhan, 1989; Novacheck, 1998) have consolidated the use of the Spring Loaded Inverted Pendulum (SLIP) to model running dynamics, which has frequently been involved in the generation of running motions for legged robots (Wensing and Orin, 2013; Secer and Saranli, 2018; Secer and Cinar, 2020). In these works, control is realized by means of a step-to-step regulation of apex states (flight states with zero vertical velocity). Xiong and Ames, (2018) derive a spring-mass model from the robot dynamics to generate hopping motions by optimizing the second-order derivative of the leg length. Winkler et al. (2018) perform offline trajectory optimization to compute dynamic motions, involving flight phases and locomotion over tilted planes. The method makes use of a convenient polynomial parameterization to efficiently optimize phase durations, foot locations, and contact forces while enforcing dynamic consistency through Centroidal Dynamics. More recently, Li et al. (2021) propose an optimization-based CoM trajectory planning using a SLIP model during stance phases for quadrupedal hopping on two legs. SLIP dynamics are highly nonlinear and thus unsuitable for predictive control unless considerably approximated.

Although the ZMP is not defined during flight phases due to the loss of contact with the ground, balance during support phases can still be enforced by means of a ZMP criterion. ZMP-based approaches for running are proposed with VH-IP modeling of the dynamics during support phases and a free-falling mass model during flight phases (Kajita et al., 2007; Takenaka et al., 2009b; Tajima et al., 2009). The vertical motion is generated independently, the ZMP trajectory is preassigned and the CoM trajectory is obtained by numerically solving the dynamics in order to meet the next step boundary conditions. Englsberger et al. (2016) generate running gaits aimed at approximating natural human behavior, using measured data and a spatially independent polynomial encoding of the CoM trajectory. More recently, Ahn and Cho, (2021) proposed an MPC formulation for executing jumping motions where the vertical trajectory is generated offline. Also, Sugihara et al. (2021) introduced another ZMP-based MPC approach to generate 3D walking and running; constraints are enforced approximately in order to obtain closed-form solutions to speed up the execution.

We recently proposed an Intrinsically Stable MPC (IS-MPC) with an explicit stability constraint embedded in the formulation (Scianca et al., 2020). This constraint is in charge of dealing with the unstable character of humanoid dynamics, by guaranteeing that the CoM trajectory is always bounded with respect to the ZMP. Since the LIP is used as a prediction model, the DCM dynamics can be decoupled by using a simple change of coordinates, and a closed-form condition on the long-term evolution can be found. Zamparelli et al. (2018) used similar ideas and proposed an IS-MPC extension that is capable of generating 3D trajectories for the CoM. This can be realized by constraining the allowed CoM evolution to a specific set of trajectories that present LIP-like dynamics along all three axes. While this approach is very effective in many circumstances, it limits the number of trajectories that can be generated and it is thus not applicable to every situation. Furthermore, it cannot be extended to generate running motions.

### 1.2 Contributions

In this article, we present a unified MPC algorithm that generates online both 3D walking and running gaits as well as transitions between them. Motions are generated in such a way to follow as closely as possible a footstep plan, in an environment constituted by horizontal patches at different heights, known as world of stairs.

The main contributions of this article can be summarized by the following points:

• we describe a scheme for generating a humanoid gait with a variable CoM height using the VH-IP model. The proposed architecture uses a two-stage MPC scheme and only requires solving standard linear-quadratic QP problems by means of the fact that the natural frequency present in the horizontal dynamics can be regarded as a time-varying parameter. With respect to the recent state of the art methods, our scheme has the advantage of maintaining a linear formulation without limiting the possible vertical CoM motions by constraining the pendulum frequency (Zamparelli et al., 2018; Sugihara et al., 2021);
• by properly shaping the vertical GRF using constraints in the first MPC stage, we introduce flight phases in order to allow the scheme to generate running motions;
• we propose a variation of our stability constraint (Scianca et al., 2020) to extend its applicability to the case of linear time-varying systems, and discuss how it is included in the formulation, both in the case of walking with variable height and running. Differently from Caron et al. (2019a), the proposed constraint is linear and can be efficiently enforced in a QP;
• we show how to extend the proposed scheme in order to allow running over tilted surfaces.

The proposed approach allows to generate gaits involving stair climbing and running for traversing complex terrain configurations. Furthermore, thanks to the ability to generate a CoM trajectory with variable height, it can realize a transition from walking to crouching (Kamioka et al., 2015), for example, in order to pass below an overhanging obstacle. The capabilities of the scheme will be demonstrated in dynamic simulations, as well as experiments for the case of walking. As a supplementary contribution, we will also present a simple footstep planner that, in response to high-level velocity commands, can be used to generate the required input for the proposed scheme.

The article is organized as follows: Section 2 describes the dynamics of balance on nonlevel ground. The VH-IP model is introduced in Section 3. The overall approach is sketched out in Section 4, and then the MPC is presented in detail in Section 5, while a simple footstep planner is discussed in Section 6. Simulations, as well as an experiment on the ROBOTIS OP3 humanoid robot, are shown in Section 7 and Section 8. In Section 9 we will briefly discuss an extension that allows the robot to run on tilted surfaces. Section 10 concludes the article.

## 2 Dynamic Balance

The ability to maintain dynamic balance is a basic requirement of any gait generation scheme. On horizontal ground, balance may be guaranteed using the ZMP criterion, which prescribes that the ZMP (the point where the horizontal component of the moment of the ground reaction forces becomes zero) should be at all times within the support polygon, that is, the convex hull of the contact surfaces.

When moving to 3D locomotion, where the contact surfaces are not necessarily coplanar, a support polygon cannot be defined. At the same time, one needs to consider that the definition of ZMP is actually satisfied along a line, and therefore this point is not necessarily located on the ground (Sardain and Bessonnet, 2004). Based on this, several authors have proposed extensions of the ZMP criterion to the 3D case; in particular, Sugihara et al. (2021) require that the ZMP belongs to a pyramid with vertex at the CoM and base defined as the convex hull of the projections of the active contact surfaces on an arbitrary plane, typically chosen as a horizontal plane below all contact surfaces.

**FIGURE 1:**
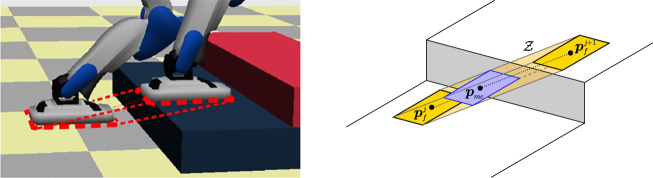
Left: In a world of stairs, where all contact surfaces are parallel to the ground, the admissible ZMP region Z in double support is an oblique prism. Right: The moving constraint region (blue) is a horizontal slice of Z (yellow) that slides between two consecutive footsteps (see Section 5.1).

In this article, we are going to consider as admissible ZMP region for balance the convex hull of the active contact surfaces:

$$Z=\left\{p:p=\overset{N_{c}}{\underset{j=1}{\sum }}\gamma_{j}v_{j},\;\text{with}\;\gamma_{j}\geq 0,\;\overset{N_{c}}{\underset{j=1}{\sum }}\gamma_{j}=1\right\},$$

where ***p*** is^2^ the generic 3D point and ***v***_*j*_, *j* = 1, *…* , *N*_*c*_, are the vertexes of all active contact surfaces. It is easy to verify that Z is a subset of the earlier-defined pyramid. In a *world of stairs*, where all contact surfaces are parallel, Z becomes an *oblique prism* in double support (see Figure 1, left). On flat ground, Z is simply reduced to the support polygon.

## 3 System Modeling

In this article, we will use the VH-IP model (Caron et al., 2019a) to characterize the relationship between the CoM and the ZMP.

**FIGURE 2:**
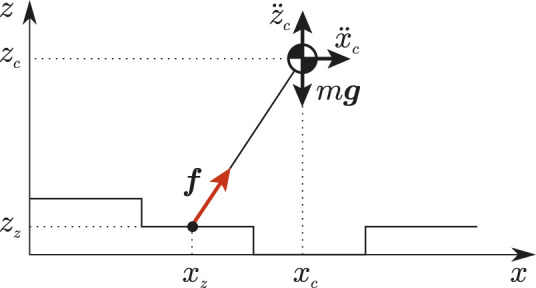
The VH-IP model in the *x*-*z* plane. Forces acting on the robot are shown in red.

Under the assumption of zero angular momentum around the CoM, the Newton and Euler (with respect to the ZMP) equations for the humanoid lead to

$$m\left({\ddot{p}}_{c}-g\right)=f,$$ (1)

$$\left(p_{z}-p_{c}\right)\times f=0,$$ (2)

where *m* is the robot mass, ***p***_*c*_ = (*x*_*c*_, *y*_*c*_, *z*_*c*_) is the CoM, ***g*** = (0, 0, − *g*) is the gravity acceleration, ***f*** is the ground reaction force (GRF in the following) and ***p***_*z*_ = (*x*_*z*_, *y*_*z*_, *z*_*z*_) is the ZMP (see Figure 2).

The CoM dynamics are deduced from Eqs (1, 2). In particular, the vertical CoM dynamics is directly given by the third equation in (1), namely

$${\ddot{z}}_{c}=\frac{f_{z}}{m}-g,$$ (3)

where *f*_*z*_ is the vertical component of the GRF. The horizontal dynamics are instead obtained as:

$$\;{\ddot{x}}_{c}=\lambda \left(x_{c}-x_{z}\right)$$ (4)

$${\ddot{y}}_{c}=\lambda \left(y_{c}-y_{z}\right),$$ (5)

where

$$\lambda =\frac{{\ddot{z}}_{c}+g}{z_{c}-z_{z}}=\frac{f_{z}}{m\left(z_{c}-z_{z}\right)}$$ (6)

represents the (variable) squared natural frequency of the pendulum.

Together, Eqs 3–5 constitute the dynamic model of our system. We consider as control inputs the horizontal ZMP components *x*_*z*_, *y*_*z,*_ and the vertical GRF *f*_*z*_, while the profile of the vertical ZMP component *z*_*z*_ is assumed to be uniquely determined^3^ once the footstep plan is specified (see Section 5.1).

Note the triangular structure of our model: the vertical dynamics (3) are independent of the horizontal dynamics (4–5). Once *f*_*z*_ is chosen, the evolution of *z*_*c*_ is determined, and the same is true for *λ*; at this point, the horizontal dynamics can be interpreted as a linear time-varying system. We will exploit this fact when designing our MPC controller.

The assumption that *f*_*z*_ is a control input requires that at least one contact with the ground is active. During flight phases, which are an integral part of a running gait, the GRF is identically zero. Setting *f* = **0** in (1) gives

$${\ddot{p}}_{c}=g,$$ (7)

indicating that the motion of the CoM is entirely controlled by gravity. Note that Eq. 7 coincides with (3–5) when *f*_*z*_ = 0.

## 4 Proposed Approach

For a humanoid robot moving in a world of stairs, we wish to design a control scheme for replicating as closely as possible an assigned footstep plan. In general, this plan will consist of a sequence of candidate footstep poses (3D positions and orientations) with specified timings. In addition, the plan also specifies for each step a reference value for the CoM height relative to the ZMP, and whether the step itself should be performed by walking or running. A simple algorithm to compute such a footstep plan will be discussed in Section 6.

**FIGURE 3:**
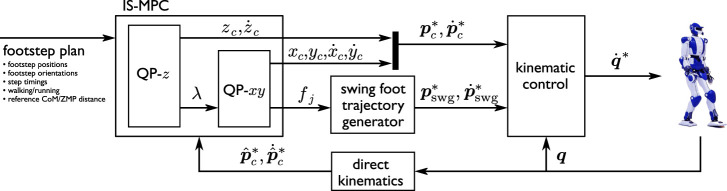
A block scheme of the proposed approach.

**FIGURE 4:**
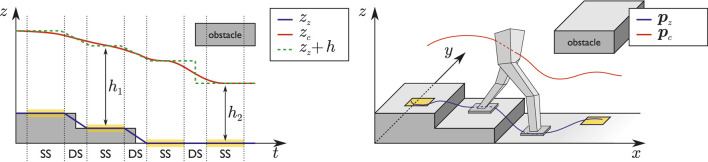
Left: a qualitative representation of the final trajectories after QP-*z* is solved in an example case. Here, the reference CoM/ZMP distance is kept constant for the initial 3 steps and then lowered in order to pass below an obstacle. Right: a qualitative representation of the trajectories generated after QP-*xy* is solved.

A block scheme of the proposed approach is shown in Figure 3. The footstep plan is the input to the IS-MPC (Intrinsically Stable MPC) block, which consists of two sequential stages. The first is a quadratic program (QP-*z*) where *f*_*z*_ in (3) is chosen over a certain control horizon to generate a vertical CoM trajectory which tracks the reference height as closely as possible. As a byproduct, the corresponding values of *λ* can be computed via (6) thanks to the triangular structure of the VH-IP model. At this point, it is possible to enter the second stage, which is another quadratic program (QP-*xy*) over the same control horizon, where *x*_*z*_, and *y*_*z*_ in (4–5) are chosen and the footsteps are adapted to generate a horizontal CoM trajectory *x*_*c*_, *y*_*c*_ resulting in a gait which is both dynamically balanced and internally stable. A qualitative representation of the trajectories produced by each stage is shown in Figure 4, with reference to a typical example.

The generated 3D CoM trajectory, along with an appropriate trajectory of the swing foot^4^, is sent to a kinematic control block where joint commands are computed using standard pseudoinversion techniques.

## 5 IS-MPC for 3D Walking and Running

In this section, we will describe in detail the IS-MPC scheme for 3D walking and running based on the VH-IP model of Section 3.

The scheme works in discrete time over time intervals of duration *δ*. A sampled variable at *t*_*k*_ will be denoted by the *k* superscript, for example, $p_{z}\left(t_{k}\right)=p_{z}^{k}$ . The control inputs of model (3–5), namely the horizontal components of the ZMP (*x*_*z*_, *y*_*z*_) and the vertical GRF *f*_*z*_, are assumed to be constant over the duration of a single interval, that is, $x_{z}\left(t\right)=x_{z}^{k}$ , $y_{z}\left(t\right)=y_{z}^{k}$ , $f_{z}\left(t\right)=f_{z}^{k}$ for $t\in \left[t_{k},t_{k+1}\right)$ .

To simplify the exposition, we will assume that all footsteps in the plan have the same orientation, taken without loss of generality to be the same of the *x* axis. This has the effect of decoupling the motion along the sagittal and coronal plane, making it possible to present the method for the *x* component only, with the understanding that a similar reasoning holds for the *y* component, except when specified. However, it is relatively easy to extend the proposed method to the case of variable orientation. When the orientation of the footsteps is not constant, the *x* and *y* components are coupled by a rotation matrix. As a consequence, a single QP problem involving both *x* and *y* components of the ZMP must be formulated, which however remains linear-quadratic provided that the orientation is not a decision variable in the QP, as shown in (Scianca et al., 2020).

**FIGURE 5:**
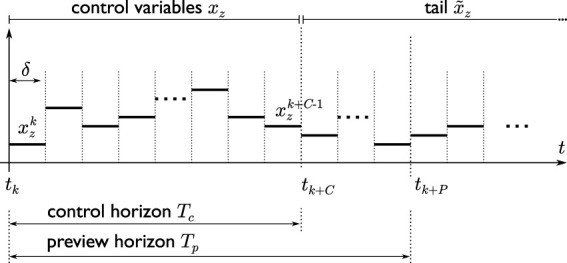
A schematic visualizing the time discretization as well as the two horizons used in the scheme.

At each time *t*_*k*_, the IS-MPC block receives the current footstep plan, that is a sequence of footstep timings $\left(t_{s}^{1},\ldots ,t_{s}^{F}\right)$ and positions $\left({\hat{x}}_{f}^{j},{\hat{y}}_{f}^{j},z_{f}^{j}\right)$ , for *j* = 1, *…* , *F*, over a *preview horizon* of duration *T*_*p*_ = *P* ⋅ *δ* (see Figure 5). Each footstep is accompanied by a reference height *h* ^*j*^ of the CoM relative to the ZMP, as well as information on whether the robot must reach the next footstep by walking or running. Note that the horizontal footstep coordinates $\left({\hat{x}}_{f}^{j},{\hat{y}}_{f}^{j}\right)$ are actually candidates which may be adapted by IS-MPC, while the vertical coordinates $z_{f}^{j}$ cannot be changed, each being the height of the corresponding ground patch. The generic step is composed by a single support phase, starting at $t_{s}^{j}$ and having duration $T_{ss}^{j}$ , followed by a double support or flight phase (depending on whether the plan specifies walking or running) for the remaining duration $t_{j+1}-t_{j}-T_{ss}^{j}$ . The number of footsteps *F* in the preview horizon will depend on the duration of each step.

On the basis of the current footstep plan, IS-MPC computes a 3D CoM trajectory and an adapted set of footsteps over a *control horizon* ^5^ *T*_*c*_ = *C* ⋅ *δ*, with *T*_*c*_ ≤ *T*_*p*_ (see Figure 5). As mentioned in the previous section, this is obtained via two sequential stages, QP-*z* and QP-*xy*. Both these programs hinge upon the definition of a suitable ZMP constraint for balance, which will therefore be introduced next.

### 5.1 Moving ZMP Constraint

As discussed in Section 2, the robot is dynamically balanced if the ZMP is inside the convex hull Z of the contact surfaces, which is an oblique prism in our world of stairs. Expressing this constraint in double support would involve products of decision variables, thus resulting in a nonlinear constraint. In order to preserve linearity, we employ a *moving constraint* (Aboudonia et al., 2017): the ZMP must belong to a fixed-shape region (the footprint) which slides between consecutive footsteps during double support and coincides with a footstep during single support. In other words, this region is a horizontal slice of the prism, see Figure 1.

**FIGURE 6:**
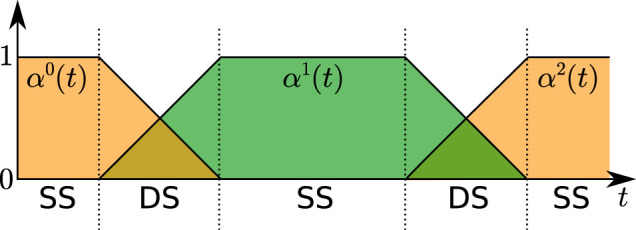
The profile of functions *α* ^*j*^(*t*) used to express the center of the moving constraint region, for a case with *F* =2. In this example, *α* ^0^(*t*) relates the center of the moving constraint to the position of the current support foot, while *α* ^1^(*t*) and *α* ^2^(*t*) relate it to the position of two predicted footsteps.

The center of the moving constraint region is denoted by $p_{mc}={\left(x_{mc}\;y_{mc}\;z_{mc}\right)}^{T}$ and is expressed as a linear function of the footstep positions:

$$p_{mc}\left(t\right)=\overset{F}{\underset{j=0}{\sum }}\alpha^{j}\left(t\right)p_{f}^{j},$$ (8)

where the *α* ^*j*^(*t*)’s are piecewise-linear functions of time, ranging between 0 and 1 and such that $\sum_{j=0}^{F}\alpha^{j}\left(t\right)=1$ , and $p_{f}^{j}={\left(x_{f}^{j}\;y_{f}^{j}\;z_{f}^{j}\right)}^{T}$ denotes the position of the *j*-th footstep. For the sake of compactness, here, we omit the expression of *α* ^*j*^(*t*), but we report a visual representation in Figure 6.

Note that, in our formulation, the moving constraint region is planar. Thus, the vertical coordinate *z*_*z*_ of the ZMP must at all times coincide with *z*_*mc*_, which is uniquely determined based on the footstep plan. In fact, Eq. 8 entails that *z*_*mc*_ only depends on the vertical coordinates of the footsteps, which are fixed and cannot be modified by IS-MPC.

### 5.2 Vertical QP

The goal of QP-*z* is to generate the vertical CoM trajectory. The prediction model is given by (3), with the decision variable *f*_*z*_ being piecewise-constant over intervals of duration *δ*:

$${\ddot{z}}_{c}=\frac{f_{z}^{k+i}}{m}-g\;\text{for}\;t\in \left[t_{k+i},t_{k+i+1}\right).\;i=0,\ldots ,C-1.$$

In order to avoid slipping while walking, the GRF must satisfy a condition of sufficient friction, commonly expressed through a friction cone (Ahn and Cho, 2021). Enforcing this directly as a constraint, however, would introduce a nonlinear coupling between the vertical GRF and the horizontal CoM accelerations. To avoid this, we impose the simplified constraint

$$f_{z}^{k+i}\geq f_{z}^{\text{min}},$$ (9)

where $f_{z}^{\text{min}}$ is a value of the vertical GRF that provides sufficient friction to avoid slipping. During single support, this can be computed by using the following relationship based on a simple Coulomb friction model:

$$f_{z}^{\text{min}}=\frac{m}{\mu }a_{\text{max}},$$

where *μ* is the friction coefficient and $a_{\text{max}}=\max\sqrt{{\ddot{x}}_{c}^{2}+{\ddot{y}}_{c}^{2}}$ is the maximum horizontal acceleration of the CoM, which can be computed in a simulated environment over a number of trials. In practice, it is observed that this value is also appropriate in double support phases.

In correspondence of flight phases (the robot is running), constraint (9) is replaced by

$$f_{z}^{k+i}=0$$ (10)

in compliance with model (7).

Collect all decision variables in a vector

$$F_{z}^{k}={\left(f_{z}^{k+1}\;\;\ldots \;\;f_{z}^{k+C-1}\right)}^{T}$$

along with the predicted state variables

$${\dot{Z}}_{c}^{k}={\left({\dot{z}}_{c}^{k}\;\;\ldots \;\;{\dot{z}}_{c}^{k+C-1}\right)}^{T}\;\;\;\;Z_{c}^{k}={\left(z_{c}^{k}\;\;\ldots \;\;z_{c}^{k+C-1}\right)}^{T}$$

and reference values for the absolute CoM height

$$Z_{c}^{k,*}={\left(z_{z}^{k}+h^{k}\;\;\ldots \;\;z_{z}^{k+C-1}+h^{k+C-1}\right)}^{T}.$$ (11)

In the latter, $z_{z}^{k+i}$ is the vertical coordinate *z*_*mc*_ of the center of the moving constraint region (see the end of Section 5.1), while *h* ^*k*+*i*^ is the reference CoM height relative to the ZMP as specified by the current footstep plan.

QP-*z* is formulated as

$$\left\{\begin{array}{c} \;\;\;\;\underset{F_{z}^{k}}{\min}\;\|Z_{c}^{k}-Z_{c}^{k,*}{\|}^{2}+\alpha_{z}\|{\dot{Z}}_{c}^{k}{\|}^{2}+\beta_{z}\|\Delta F_{z}^{k}{\|}^{2}\;\\ subject\;to:\;\\ •\;constraint\left(9\right),for\;each\;interval\;\left[t_{k+i},t_{k+i+1}\right]\;where\;the\;robot\;is\;walking\;\\ •\;constraint\left(10\right),for\;each\;interval\;\left[t_{k+i},t_{k+i+1}\right]\;where\;the\;robot\;is\;running,\; \end{array}\right.$$

where *α*_*z*_, *β*_*z*_ are positive weights. The first term of the cost function takes care of tracking the reference CoM height, while the second term penalizes sudden height variations. The last term is included to reduce the difference between consecutive samples of the vertical GRF, so as to generate a smoother GRF profile.

As discussed in Section 4, the vertical GRF produced in output by QP-*z* is not directly applied to the system. Instead, the associated vertical CoM trajectory will be sent to the kinematic controller and converted to suitable joint commands for the robot.

### 5.3 Horizontal QP

The goal of QP-*xy* is to complete the generation of the CoM trajectory with the horizontal components. Thanks to the assumption that all footsteps have the same orientation, QP-*xy* itself can be split into two decoupled programs QP-*x* and QP-*y*. We will present in detail the formulation of QP-*x*, with the understanding that QP-*y* is formally identical except when explicitly noted.

When the robot is walking, its CoM obeys the horizontal dynamics (4–5), where the time-varying *λ* should be computed via (6) using the vertical CoM trajectory generated by QP-*z*. In particular, we can sample-and-hold *λ*(*t*) to obtain a piecewise-time-invariant prediction model:

$${\ddot{x}}_{c}=\lambda^{k+i}\left(x_{c}-x_{z}\right)\;\text{for}\;t\in \left[t_{k+i},t_{k+i+1}\right),\;i=0,\ldots ,C-1,$$ (12)

where

$$\lambda^{k+i}=\frac{{\ddot{z}}_{c}^{k+i}+g}{z_{c}^{k+i}-z_{z}^{k+i}}.$$

In time intervals where the robot is running, its CoM obeys the flight-phase dynamics (7), and thus the prediction model becomes

$${\ddot{x}}_{c}=0,$$

which, as already noticed, is equivalent to setting *λ* ^*k*+*i*^ = 0 in (12).

QP-*x* will enforce five kinds of constraints, namely: ZMP constraints, ground patch constraints, footstep kinematic constraints, swing foot constraints, and finally a special stability constraint.

#### 5.3.1 ZMP Constraints

Assume^6^ the footprints to be rectangular regions with dimensions *d*_*z*,*x*_ and *d*_*z*,*y*_. Following the discussion in Section 5.1, in a generic time interval we guarantee balance by confining the ZMP to a moving constraint region having the same shape:

$$-\frac{1}{2}\left(\begin{array}{c} d_{z,x}\\ d_{z,y} \end{array}\right)\leq \left(\begin{array}{c} x_{z}^{k+i}\\ y_{z}^{k+i} \end{array}\right)-\left(\begin{array}{c} x_{mc}^{k+i}\\ y_{mc}^{k+i} \end{array}\right)\leq \frac{1}{2}\left(\begin{array}{c} d_{z,x}\\ d_{z,y} \end{array}\right),$$ (13)

where the coordinates (*x*_*mc*_, *y*_*mc*_) of the moving constraint region are given by (8).

#### 5.3.2 Ground Patch Constraint

**FIGURE 7:**
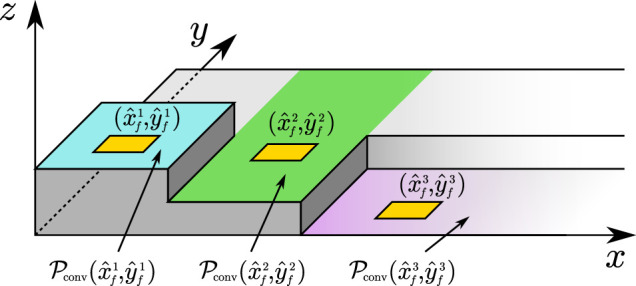
A visual representation of the convex regions used in the ground patch constraint.

To maintain linearity of the constraints, we do not allow footstep adaptations that would move the footstep to a ground patch located at a different height from that of the candidate footsteps. For this reason, we enforce the following constraint:

$$\left(x_{f}^{j},y_{f}^{j}\right)\in P_{\text{conv}}\left({\hat{x}}_{f}^{j},{\hat{y}}_{f}^{j}\right)⊖P_{\text{foot}},$$ (14)

where $P_{\text{conv}}\left(x,y\right)$ is a convex region that includes point (*x*, *y*) and is contained in a single ground patch, ⊖ denotes a Minkowski difference (Montejano, 1996), and $P_{\text{foot}}=\left\{\left(x,y\right):|x|\leq d_{z,x}/2,|y|\leq d_{z,y}/2\right\}$ is a rectangular region having the same dimensions of the footprint, see Figure 7. It is clearly beneficial to choose $P_{\text{conv}}\left(x,y\right)$ as large as possible.

In this article, we assume the full knowledge of the environment and therefore the above computation can also be performed offline. For algorithmic solutions to this problem see, for example, (Deits and Tedrake, 2014; Deits and Tedrake, 2015).

#### 5.3.3 Kinematic Constraints

The third type of constraint ensures that footsteps are placed so as to be kinematically realizable by the robot. This means enforcing a box constraint on the step length:

$$\left|x_{f}^{j}-x_{f}^{j-1}\right|\leq \frac{d_{a,x}\left(z_{f}^{j-1},z_{f}^{j}\right)}{2},\;\;\;\;\;\left|y_{f}^{j}-y_{f}^{j-1}\pm \ell \right|\leq \frac{d_{a,y}\left(z_{f}^{j-1},z_{f}^{j}\right)}{2},$$ (15)

with $d_{a,x}\left(z_{f}^{j-1},z_{f}^{j}\right)$ and $d_{a,y}\left(z_{f}^{j-1},z_{f}^{j}\right)$ denoting the dimensions of the admissible region along *x* and *y*, *ℓ* is an average coronal displacement between consecutive footsteps, and the plus/minus signs alternates between left and right footsteps. Note that $d_{a,x}\left(z_{f}^{j-1},z_{f}^{j}\right)$ and $d_{a,y}\left(z_{f}^{j-1},z_{f}^{j}\right)$ are assumed to be functions of $z_{f}^{j-1}$ and $z_{f}^{j}$ because they should depend on the difference in height between the two corresponding patches. Intuitively, climbing higher stairs limits the horizontal leg extension; we encode this idea by letting

$$d_{a,x}\left(z_{f}^{j-1},z_{f}^{j}\right)=\left(1-\sigma \frac{\left|z_{f}^{j}-z_{f}^{j-1}\right|}{\Delta z_{f}^{\text{max}}}\right)d_{a,x}^{0},\;d_{a,y}\left(z_{f}^{j-1},z_{f}^{j}\right)=\left(1-\sigma \frac{\left|z_{f}^{j}-z_{f}^{j-1}\right|}{\Delta z_{f}^{\text{max}}}\right)d_{a,y}^{0},$$

where *σ* ∈ (0, 1), $\Delta z_{f}^{\text{max}}$ is the maximum height variation that the robot can realize, and $d_{a,x}^{0},d_{a,y}^{0}$ are the dimensions of the admissible region on planar ground. Note that (15) is the only constraint whose expression differs between QP-*x* and QP-*y*, due to the inclusion of the lateral displacement *ℓ*.

#### 5.3.4 Swing Foot Constraint

To prevent the leg joint speed from exceeding a feasible range during the swing foot motion, we enforce the following swing foot constraint (Herdt et al., 2010)

$$\left|x_{\text{sw}}^{k}-x_{f}^{1}\right|\leq \left(t_{s}^{0}+T_{ss}^{0}-t_{k}\right)\;v_{\text{sw}}^{\text{max}},$$ (16)

where $t_{s}^{0}$ is the starting time of the current step, $T_{ss}^{0}$ the single support duration and the quantity $\left(t_{s}^{0}+T_{ss}^{0}-t_{k}\right)$ represents the remaining time to complete the current single support phase. This linear constraint prevents infeasible leg motions for the humanoid by increasingly limiting the distance of the next footstep $x_{f}^{1}$ from the current swing foot position $x_{\text{sw}}^{k}$ as the foot is landing. The velocity bound term $v_{\text{sw}}^{\text{max}}$ can be computed from the maximum foot Cartesian velocity that the robot can realize. A similar constraint must be enforced for the *y* component.

#### 5.3.5 Stability Constraint

All models of humanoid robot dynamics have an underlying unstable behavior, due to the balancing nature of these systems. As a consequence, keeping the ZMP inside the support polygon is not sufficient to generate a successful gait. In fact, a perfectly acceptable ZMP trajectory could be associated with a diverging CoM trajectory, making the resulting motion infeasible for the robot in practice. The goal of this section is to derive a constraint capable of guaranteeing the internal stability of the scheme, that is, keeping the CoM trajectory bounded with respect to the ZMP.

In previous works (Scianca et al., 2016; Scianca et al., 2020), we approached the above problem by enforcing a *stability condition* for the LIP model, in which *z*_*c*_ − *z*_*z*_ is assumed to be constant. Let us consider the same model as a starting point:

$${\ddot{x}}_{c}=\lambda_{\text{LIP}}\left(x_{c}-x_{z}\right),$$ (17)

where *λ*_LIP_ = *g*/(*z*_*c*_ − *z*_*z*_) is constant. Using the new coordinate

$$x_{u}=x_{c}+\frac{{\dot{x}}_{c}}{\sqrt{\lambda_{\text{LIP}}}},$$ (18)

the unstable dynamics is isolated and expressed as:

$${\dot{x}}_{u}=\sqrt{\lambda_{\text{LIP}}}\left(x_{u}\;-\;x_{z}\right),$$ (19)

with $\sqrt{\lambda_{\text{LIP}}}$ the natural frequency of the equivalent pendulum.

The instability of system (17) implies that, for a generic ZMP trajectory, *x*_*u*_ would diverge with respect to *x*_*z*_, even if the ZMP is inside the support polygon. However, at time *t*_*k*_, the system can be initialized in such a way that the free evolution of (19) exactly cancels the component of the forced evolution that would diverge with respect to *x*_*z*_, allowing the CoM trajectory to remain bounded with respect to the ZMP. This special initialization depends on the future ZMP trajectory, and is identified by the following proposition.

Proposition 1 Assume that |*x*_*z*_ (*t*′) − *x*_*z*_(*t*)| ≤ *a* + *b* (*t*′ − *t*), *∀t*′ ≥ *t*, for some *a*, *b* > 0, and that

$$x_{u}\left(t_{k}\right)=\sqrt{\lambda_{\text{LIP}}}\int_{t_{k}}^{\infty }e^{-\sqrt{\lambda_{\text{LIP}}}\left(\tau -t_{k}\right)}x_{z}\left(\tau \right)d\tau ,$$ (20)

then, system (17) is internally stable, that is, *x*_*c*_ is bounded with respect t*o* *x*_*z*_:

$$\exists M>0:\left|x_{c}\left(t\right)-x_{z}\left(t\right)\right|\leq M,\;t\geq t_{k}.$$

*Proof*. See Supplementary Appendix S1.

The first hypothesis in this proposition requires that the future ZMP trajectory is bounded by a linear function. This allows the ZMP to be discontinuous, but it does not allow arbitrarily large skips. Note that this requirement is in our case always satisfied due to the presence of the kinematic constraint. In fact, a bounding linear function for the future ZMP can easily be derived by considering the maximum allowed step size and the step duration. We omit however the details of this calculation as it is fairly tedious, and the specific expression of this bound offers no additional insight.

This result is not directly applicable to the present setting, because system (12) is time-varying and therefore the coordinate change (18) does not actually isolate the unstable dynamics. To recover a favorable situation, we will assume in our prediction model that *λ* ^*k*+*i*^ = *λ*_LIP_ for *t* ≥ *t*_*k*+*C*_, consistently with the fact that the control horizon does not extend beyond *t*_*k*+*C*_. This is equivalent to defining a modified prediction model alternative to (12) given by

$${\ddot{x}}_{c}=\left\{\begin{array}{cc} \lambda^{k+i}\left(x_{c}-x_{z}\right)\; & \text{for}\;t\in \left[t_{k+i},t_{k+i+1}\right),\;i=0,\ldots ,C-1\\ \lambda_{\text{LIP}}\left(x_{c}-x_{z}\right)\; & \text{for}\;t\geq t_{k+C}, \end{array}\right.$$ (21)

with $\lambda_{\text{LIP}}=g/\left(x_{z}^{k+C}+h^{k+C}\right)$ . Note that (21) is identical to the time-varying (12) system up to the horizon *t*_*k*+*C*_ and becomes time-invariant after that.

For system (21) we can formulate a stability condition, as expressed by the following:

Proposition 2 Assume that |*x*_*z*_ (*t*′) − *x*_*z*_(*t*)| ≤ *a* + *b* (*t*′ − *t*)*,* *∀t*′ ≥ *t* *,* for some *a*, *b* > 0*,* and that the current state $\left(x_{c}^{k},{\dot{x}}_{c}^{k}\right)$ satisfies

$$G\;\Phi \left(t_{k+C},t_{k}\right)\left(\begin{array}{c} x_{c}^{k}\\ {\dot{x}}_{c}^{k} \end{array}\right)+\int_{t_{k}}^{t_{k+C}}G\;\Phi \left(t_{k+C},\tau \right)B\left(\tau \right)x_{z}\left(\tau \right)d\tau =x_{u}^{k+C},$$ (22)

where $G=\left(1\;1/\sqrt{\lambda_{\text{LIP}}}\right)$ , the terms Φ(*t*_*k*+*C*_, *t*) and *B*(*t*) are respectively the transition and the input matrix for system (12)*,* and

$$x_{u}^{k+C}=\sqrt{\lambda_{\text{LIP}}}\int_{t_{k+C}}^{\infty }e^{-\sqrt{\lambda_{\text{LIP}}}\left(\tau -t_{k+C}\right)}x_{z}\left(\tau \right)d\tau .$$ (23)

Then*,* system (21) is internally stable, that is, *x*_*c*_ is bounded with respect to *x*_*z*_ *:*

$$\exists M>0:\left|x_{c}\left(t\right)-x_{z}\left(t\right)\right|\leq M,\;t\geq t_{k}.$$

*Proof.* System (21) is equivalent to system (17) for *t* ≥ *t*_*k*+*C*_. Hence, Prop. 1 states that if $x_{u}^{k+C}$ is given by (23), then the subsequent evolution of *x*_*c*_ will be bounded with respect to *x*_*z*_. On the other hand, it is $x_{u}^{k+C}=G\;{\left(x_{c}^{k+C}\;\;{\dot{x}}_{c}^{k+C}\right)}^{T}$ , with

$$\left(\begin{array}{c} x_{c}^{k+C}\\ {\dot{x}}_{c}^{k+C} \end{array}\right)=\Phi \left(t_{k+C},t_{k}\right)\left(\begin{array}{c} x_{c}^{k}\\ {\dot{x}}_{c}^{k} \end{array}\right)+\int_{t_{k}}^{t_{k+C}}\Phi \left(t_{k+C},\tau \right)B\left(\tau \right)x_{z}\left(\tau \right)d\tau .$$

The thesis follows immediately.

Condition (22) is noncausal, as it depends on the entire future ZMP trajectory. To find a causal condition we proceed, similarly to (Scianca et al., 2020), by conjecturing an anticipative tail, that is, a ZMP trajectory ${\tilde{x}}_{z}\left(t\right)$ for $t\in \left[t_{k+C},\infty \right)$ , on the basis of the available preview information from the footstep plan. In particular, this trajectory coincides with the center of the future moving constraint region

$${\tilde{x}}_{z}\left(t\right)=\left\{\begin{array}{cc} \; & x_{mc}^{k+i}\;\;\text{for}\;t\in \left[t_{k+i},t_{k+i+1}\right),\;i=C,\ldots ,P-1\\ \; & x_{mc}^{k+P}\;\text{for}\;t\in \left[t_{k+P},\infty \right), \end{array}\right.$$

where $x_{mc}^{k+i}$ is computed from (8). At this point, one obtains a causal version of (22) by replacing *x*_*z*_ with ${\tilde{x}}_{z}$ in (23), obtaining

$$G\;\Phi \left(t_{k+C},t_{k}\right)\left(\begin{array}{c} x_{c}^{k}\\ {\dot{x}}_{c}^{k} \end{array}\right)+\int_{t_{k}}^{t_{k+C}}G\;\Phi \left(t_{k+C},\tau \right)B\left(\tau \right)x_{z}\left(\tau \right)d\tau =\sqrt{\lambda_{\text{LIP}}}\int_{t_{k+C}}^{\infty }e^{-\sqrt{\lambda_{\text{LIP}}}\left(\tau -t_{k+C}\right)}{\tilde{x}}_{z}\left(\tau \right)d\tau .$$ (24)

To convert this causal condition into a constraint, it is necessary to express the integral in the left hand side in terms of the MPC decision variables, that is the samples of the piecewise-constant ZMP. This requires explicitly stating the form of the state transition matrix Φ(*t*_*k*+*C*_, *t*) and the input matrix *B*(*t*). In particular, it is sufficient to specify these terms at each sampled time instant *t*_*k*+*i*_ along the control horizon, recalling that their expression will vary depending on whether *t*_*k*+*i*_ belongs to a support or a flight phase. The state transition matrix from *t*_*k*+*i*_ to *t*_*k*+*C*_ is given by:

$$\Phi \left(t_{k+C},t_{k+i}\right)=\overset{C-1}{\underset{j=i}{\prod }}\Phi_{k+j}\;i=0,\ldots ,C-1,$$

where in support and flight phases we have, respectively,

$$\Phi_{k+j}=\left(\begin{array}{cc} \cosh\left(\sqrt{\lambda^{k+j}}\delta \right) & \frac{\sinh\left(\sqrt{\lambda^{k+j}}\delta \right)}{\sqrt{\lambda^{k+j}}}\\ \sinh\left(\sqrt{\lambda^{k+j}}\delta \right)\sqrt{\lambda^{k+j}} & \cosh\left(\sqrt{\lambda^{k+j}}\delta \right) \end{array}\right)\;\text{and}\;\Phi_{k+j}=\left(\begin{array}{cc} 1 & \delta \\ 0 & 1 \end{array}\right).$$

Similarly, for the input matrix we have, respectively,

$$B\left(t_{k+i}\right)=\left(\begin{array}{c} 1-\cosh\left(\sqrt{\lambda^{k+i}}\delta \right)\\ -\sinh\left(\sqrt{\lambda^{k+i}}\delta \right)\sqrt{\lambda^{k+i}} \end{array}\right)\;\text{and}\;B\left(t\right)=\left(\begin{array}{c} 0\\ 0 \end{array}\right)$$

in support and flight phases.

Substituting these into (24) leads to the stability constraint

$$G\overset{C-1}{\underset{i=0}{\sum }}\overset{C-1}{\underset{j=i}{\prod }}\Phi_{k+j}B_{k+i}x_{z}^{k+i}=\sqrt{\lambda_{\text{LIP}}}\int_{t_{k+C}}^{\infty }e^{-\sqrt{\lambda_{\text{LIP}}}\left(\tau -t_{k+C}\right)}{\tilde{x}}_{z}\left(\tau \right)d\tau -G\overset{C-1}{\underset{i=0}{\prod }}\Phi_{k+i}\left(\begin{array}{c} x_{c}^{k}\\ {\dot{x}}_{c}^{k} \end{array}\right).$$

#### 5.3.6 QP-*x* Formulation

Collect the candidate footstep coordinates over the control horizon in vector

$${\hat{X}}_{f}^{k}={\left({\hat{x}}_{f}^{1}\;\ldots \;{\hat{x}}_{f}^{F}\right)}^{T}$$

and the decision variables in vectors

$$X_{z}^{k}={\left(x_{z}^{k}\;\;\ldots \;\;x_{z}^{k+C-1}\right)}^{T}\;X_{f}^{k}={\left(x_{f}^{1}\;\ldots \;x_{f}^{F}\right)}^{T}.$$

Moreover, define $X_{mc}^{k}={\left(x_{mc}^{k}\;\;\ldots \;\;x_{mc}^{k+C-1}\right)}^{T}$ as a vector containing the *x* coordinate of the center of the moving constraint region (8). Finally, define the ZMP increment vector as $\Delta X_{z}={\left(x_{z}^{k+1}-x_{z}^{k}\;\ldots \;x_{z}^{k+C}-x_{z}^{k+C-1}\right)}^{T}$ .

QP-*x* is formulated as:

$$\left\{\begin{array}{c} \;\;\;\underset{X_{z}^{k},X_{f}^{k}}{\min}\;\|X_{z}-X_{mc}^{k}{\|}^{2}+\alpha_{x}\|\Delta X_{z}{\|}^{2}+\beta_{x}\|X_{f}^{k}-{\hat{X}}_{f}^{k}{\|}^{2}\;\\ subject\;to:\;\\ •\;ZMP\;constraints\;\left(13,x\;component\right),\;for\;j=0,\ldots ,F\;\;\\ •\;ground\;patch\;constraints\;\left(14,\;x\;component\right),\;for\;j=1,\ldots ,P\;\\ •\;kinematic\;constraints\;\left(15,\;x\;component\right),\;for\;j=1,\ldots ,F\;\\ •\;swing\;foot\;constraint\;\left(16\right)\;\\ •\;stability\;constraint\;(22).\; \end{array}\right.$$

The first term of the cost function depends on all decision variables, directly on the ZMP and indirectly on the footsteps (through $X_{mc}^{k}$ ); its inclusion keeps the ZMP as much as possible close to the center of the moving constraint region. The second term minimizes ZMP variations in order to increase the smoothness of the resulting trajectory. The third term aims at realizing the candidate’s footstep sequence as closely as possible.

**FIGURE 8:**
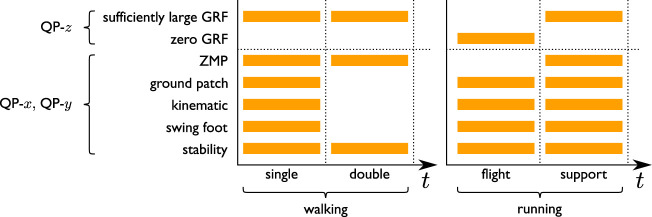
Activation of the various constraints during a *i*) walking and *ii*) running step.

An analogous problem QP-*y* is formulated for the *y* component, the only difference being that the kinematic constraint is given by the second expression in (15). Note that the ZMP constraints are obviously not enforced in time intervals belonging to flight phases; for the same reason, no ZMP decision variables are associated with such intervals. The constraints on the footstep placement are instead activated or deactivated depending on the gait type (walking/running) and the specific phase (single/double support, support/flight). Figure 8 recapitulates this alternation.

The first ZMP sample $\left(x_{z}^{k},y_{z}^{k}\right)$ resulting from QP-*x* and QP-*y* is used in the prediction model to compute the next sample of the horizontal CoM trajectory $\left(x_{c}^{k+1},y_{c}^{k+1}\right)$ . Together with the result of QP-*z*, this gives the complete sample of the CoM trajectory. The first footstep position $x_{f}^{1},y_{f}^{1}$ is instead used as target landing position for the swing foot in the next single support phase. CoM and swing foot trajectories are then tracked by the kinematic controller.

We conclude this section with a comment regarding the feasibility of QP-*x* and QP-*y*. Even if the stability constraint impacts the feasibility of the scheme (a detailed analysis for the planar walking case has been provided in Scianca et al. (2020)), there can be situations in which other constraints may conflict. For instance, reaching the assigned ground patch could conflict with the kinematic constraints if a strong deviation from the footstep plan has been previously required; in these situations, the horizontal QP can become unfeasible. This is a limitation that can be overcome by using a motion replanner rather than just relying on the local footstep adjustment capabilities of the controller.

## 6 A Velocity-Driven Footstep Planner

This section describes a possible method for determining the required inputs for the proposed scheme in response to a high-level forward velocity command. We assume that the 3D map of the environment, called “ elevation map”, is known. In addition to determining footstep placements and timing, we propose a simple criterion to decide for each step whether it will be realized by walking or running, and the corresponding reference CoM height relative to the ZMP.

The basic idea is that a change in velocity should be reflected in a change of both the spacing between the footsteps and their timing (Scianca et al., 2020). The goal is, given a desired velocity *v*, to determine a step length *L* and a step duration *T* such that *L*/*T* = *v*. The method assumes that a triplet of cruise parameters $\left(\bar{v},\bar{L},\bar{T}\right)$ is available, where $\bar{v}=\bar{L}/\bar{T}$ , with $\bar{L}$ and $\bar{T}$ denoting the typical step length and duration for the robot under consideration (for instance, the parameters of the default walking gait implemented by the robot manufacturer).

A deviation from $\bar{v}$ should have an effect both on the step length and duration, which is expressed as:

$$v=\bar{v}+\Delta v=\frac{\bar{L}+\Delta L}{\bar{T}-\Delta T}.$$

By setting Δ*L* = *α*Δ*T* with *α* > 0, one can derive an expression for the step duration realizing *v*

$$T=\bar{T}\;\frac{\alpha +\bar{v}}{\alpha +v},$$

which has to be divided into phases (single/double or support/flight). Such division can be directly chosen based on typical values for the ratio between phase durations in human walking and running. As a general rule based on human biomechanics (Novacheck, 1998), the double support duration should constitute the 20–40% of the step duration. Although this proportion should be inverted for flight phases during the run, here we will use for simplicity the same proportion in running gaits. The associated step length is easily calculated as *L* = *vT*.

This procedure outputs a larger *L* if a larger velocity *v* is commanded. It stands to reason that shorter steps should be realized by walking, whereas larger steps require running. In practice one may identify a step length threshold *L*_max_ and command the scheme to generate a running step (mode = run) when *L* > *L*_max_, and a walking step (mode = walk) otherwise. Being this simple planner conceived for reference forward velocity tracking, the candidate’s footsteps on the *y* direction simply alternate with a fixed foot distance with respect to the initial CoM position.

So far, a triplet $\left(L^{j},T^{j},{mode}^{j}\right)$ has been generated for the *j*-th step in the preview horizon, from which it is immediate to compute the horizontal positions $\left({\hat{x}}_{f}^{j},{\hat{y}}_{f}^{j}\right)$ of the candidate footsteps. In order to generate the vertical part of the plan, we need to retrieve from the elevation map the ground patch $P_{\text{conv}}\left({\hat{x}}_{f}^{j},{\hat{y}}_{f}^{j}\right)$ corresponding to each footstep. If the footstep is unfeasible (e.g., because it is not completely contained in the patch), it is simply translated towards the previous footstep until feasibility is recovered. More sophisticated rules can be introduced, for example, enforcing a step with zero sagittal strides each time the two footsteps are located at two different heights^7^.

Finally, we need to assign the reference CoM height relative to the ZMP during the step, which will be used in (11). In particular, we choose a constant reference value $h^{j}=\bar{h}$ throughout the gait. This value can be modified if necessary, for example, to pass below an obstacle by crouching, as will be shown in the next result sections.

## 7 Simulations

In order to validate the proposed approach, we now present dynamic simulations on an HRP-4 humanoid robot in the DART dynamic environment. The actual torque limits of the platforms were not considered, as the primary objective here is to demonstrate the dynamic stability of a physically simulated robot performing dynamic gaits generated by our IS-MPC scheme. It should be noted in the fact that at the time of conducting this research, just a small percentage of the currently available humanoid robots would be able to perform dynamic running in a 3D environment, for example, Atlas by Boston Dynamics or ongoing projects such as the MIT humanoid (Chignoli et al., 2021) and Kangaroo by PAL Robotics^8^.

In all the simulations the kinematic controller runs at 100 Hz, while the vertical and horizontal QPs are solved using *hpipm* (Frison and Diehl, 2020). The simulations use the following parameters: sampling time *δ* = 0.01 s, control horizon *T*_*c*_ = 0.7 s, preview horizon *T*_*p*_ = 1.8 s, $f_{z}^{\text{min}}=114$ N, dimensions of the moving constraint region *d*_*z*,*x*_ = *d*_*z*,*y*_ = 0.08 m, dimensions of the kinematically admissible region *d*_*a*,*x*_ = 1 m, *d*_*a*,*y*_ = 0.12 m, with lateral displacement *ℓ* = 0.25 m. The cost function weights of QP-*z*, QP-*x* and QP-*y* are, respectively, *α*_*z*_ = *β*_*z*_ = 10^–5^, *α*_*x*_ = *α*_*y*_ = 1, and *β*_*x*_ = *β*_*y*_ = 10^4^.

Clips of all simulations are included in Supplementary Video S1.

### 7.1 Walking on Flat Ground With Variable CoM Height

For this simulation, the ground is flat and therefore considered as a single horizontal patch. The candidate footstep positions are equally spaced by 0.15 m along *x*, and displaced by ± 0.18 m on *y*. Every step lasts 0.7 s, divided in 0.5 s for the single support and 0.2 s for the double support. The desired vertical CoM/ZMP displacement *h* varies throughout the simulation. It is initially 0.7 m for the first four steps, then is raised to 0.77 m for four subsequent steps. At each of the following five steps, it is progressively decreased by 0.02 m until reaching the final value of 0.58 m.

**FIGURE 9:**
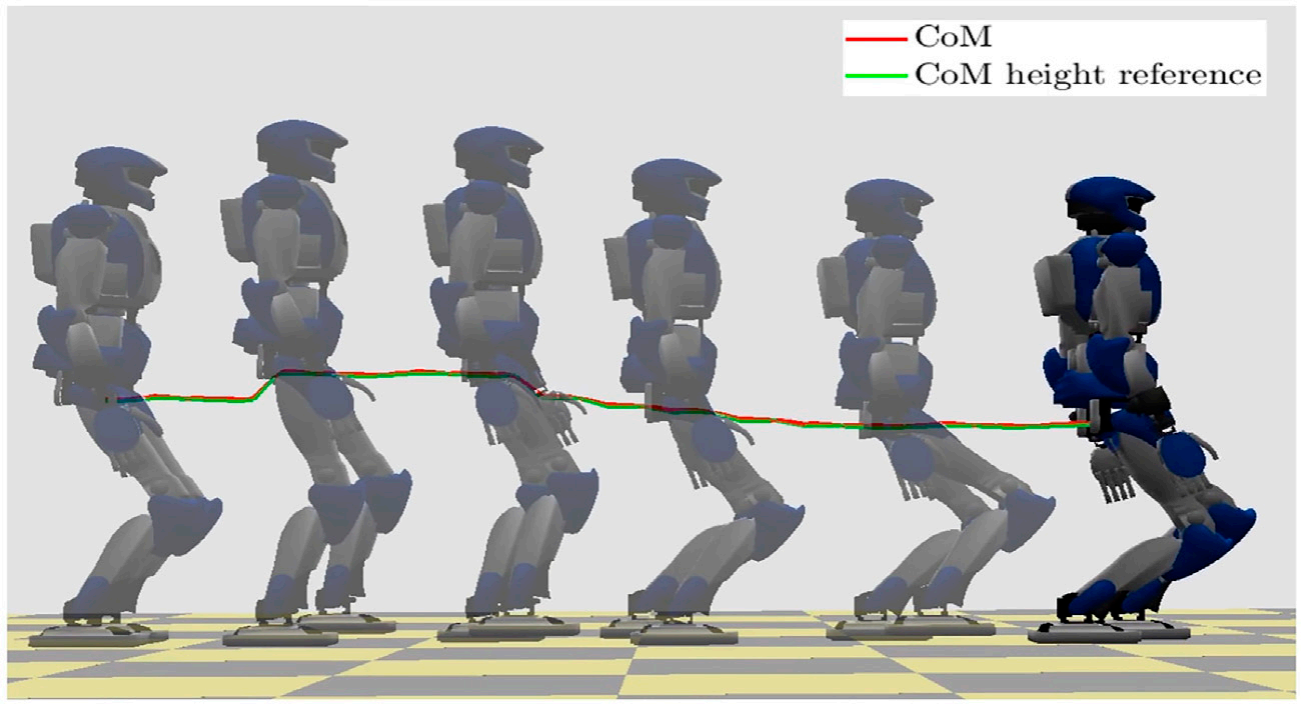
Walking on flat ground with variable CoM height: stroboscopic motion.

**FIGURE 10:**
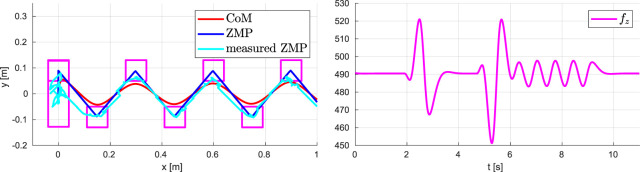
Walking on flat ground with variable CoM height: CoM/ZMP trajectories (left) and vertical GRF (right).

The simulation results are reported in Figures 9, 10. The stroboscopic motion of Figure 9 also shows the actual CoM trajectory; note the tracking of the variable reference height. In the first plot of Figure 10, we display the CoM and ZMP trajectory in the (*x*, *y*) plane for the first eleven seconds of the gait. The CoM trajectory is clearly bounded with respect to the ZMP, proving the effectiveness of the proposed stability constraint. The second plot reports the vertical GRF computed by QP-*z*, which generates the variations of the CoM height. Note its smooth profile which is due to the last term of the cost function of QP-*z*. See the Supplementary Video S1.

### 7.2 Running on Flat Ground

For the running case, the spacing of the candidate footsteps along *x* is increased to 0.3 m, and the step duration is now 0.3 s for the single support phase and 0.15 s for the flight phase. At the start, an initial walking step is performed in preparation for running. The desired vertical CoM/ZMP displacement is kept constant at *h* = 0.7 m. The arm swing is enforced by a dedicated arm controller; its frequency is the same as the gait, while the amplitude is empirically assigned, similar to what was done in Kajita et al. (2002).

**FIGURE 11:**
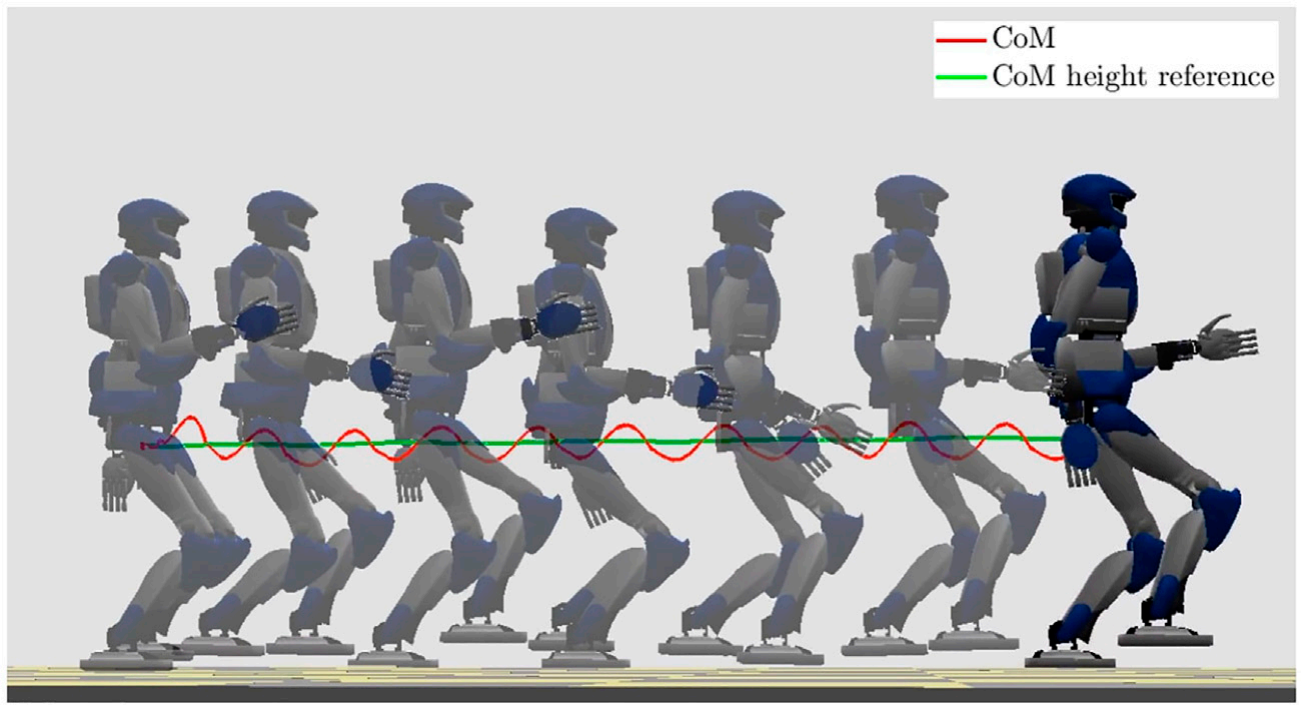
Running on flat ground: stroboscopic motion.

**FIGURE 12:**
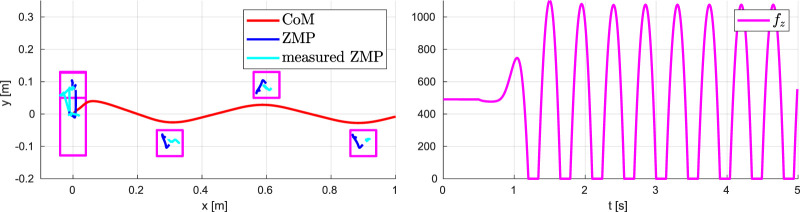
Running on flat ground: CoM/ZMP trajectories (left) and vertical GRF (right).

The results are shown in Figures 11, 12. The vertical CoM motion clearly exhibits the typical behavior observed in human biomechanics (Blickhan, 1989; Novacheck, 1998), consisting of the alternation between *absorption* and a *generation* phase: absorption is characterized by a horizontal deceleration and vertical descent of the CoM, while during the generation phase the CoM accelerates horizontally and rises in height until reaching an apex. Figure 12 shows that the ZMP trajectory is discontinuous (consistent with the fact that the ZMP is not defined during the flight phases) and the CoM trajectory is bounded. The vertical GRF has a smooth profile while presenting small jumps when starting or concluding a support phase, due to constraints (9). However, the last term in the cost function of QP-z pushes the vertical GRF at the transition towards the minimum admissible value, which in itself is quite low with respect to the GRF peaks during running.

Overall, we were able to reach a maximum forward velocity of 1.25 m/s. In our opinion, this value is due to: *i*) the simplified model and *ii*) the kinematic controller. The effect of the simplified model could be alleviated by using a stabilizer based on the ZMP feedback. As for the kinematic controller, this can be improved by properly addressing the foot impact phase.

The results are illustrated in more detail in Supplementary Video S1, in which we also included a push recovery simulation to further show the versatility of the scheme. In this simulation, HRP-4 is pushed twice by forces lasting 0.1 s of (50, 75, 0) N and (0, 75, 0) N, respectively. By adapting the footstep positions the robot can withstand the disturbances and proceed with the running gait. The same pushes would destabilize the robot if step adaptation was removed.

### 7.3 Walking and Running in a World of Stairs

In this simulation, the robot moves in a world of stairs. An additional overhanging obstacle was placed along the path, which requires the robot to lower its CoM in order to pass below it. The footstep plan is generated by the planner described in Section 6, where we have set *α* = 0.4, $\bar{T}=0.9$ s, $\bar{L}=0.3$ m, and *L*_max_ = 0.35 m. The reference velocity *v* is initially chosen as 0.3 m/s and then increased to 1.15 m/s after 16 s. The ground patches are assigned offline based on the elevation map as well as the reference CoM height, which is locally modified in order to let the robot pass below the overhanging obstacle.

**FIGURE 13:**
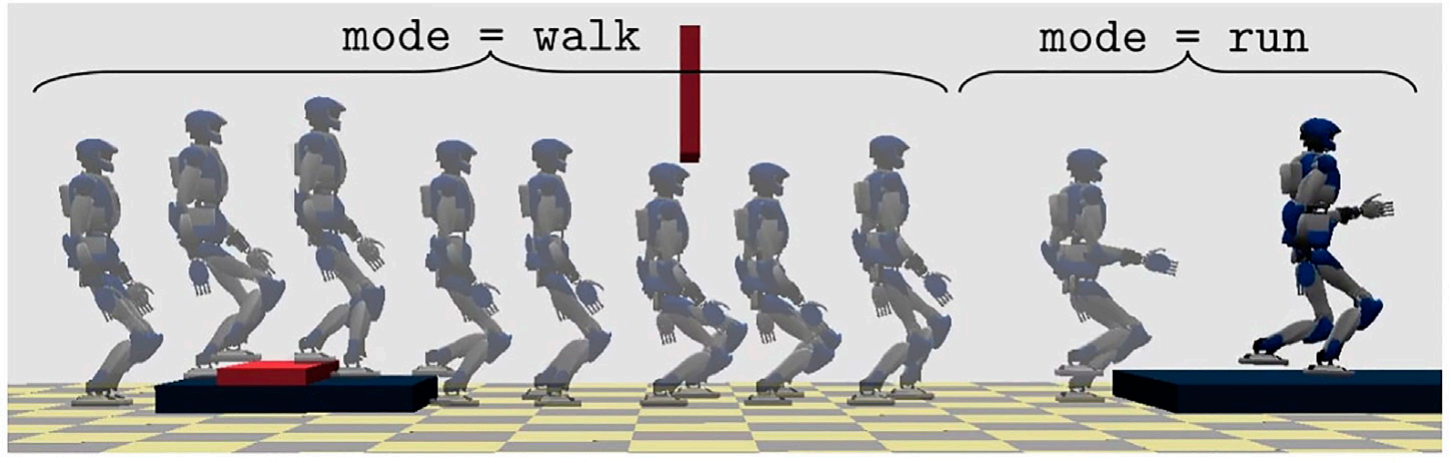
Walking and running in a world of stairs: stroboscopic motion.

**FIGURE 14:**
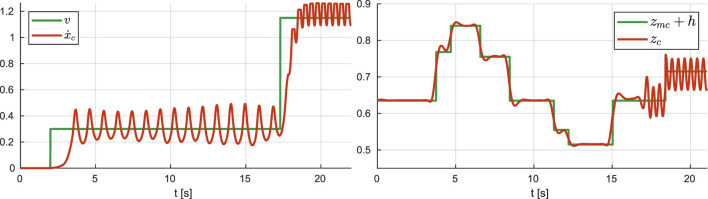
Walking and running in a world of stairs: reference and actual sagittal velocity (left), reference and actual CoM height (right).

Simulation results are reported in Figures 13, 14. The robot starts walking (mode = walk) with a step timing of 0.94 s (divided in 0.74 s for the single support phase and 0.2 s for the double support phase) and a step length along the sagittal direction of 0.28 m. When the reference velocity changes, the step length increases by 0.48 m, triggering a transition to running (mode = run); the corresponding durations are 0.24 s for the single support phase and 0.2 s for the flight phase. The sagittal velocity plot shows a satisfactory tracking of the reference sagittal velocity, while the CoM height follows the ground patch profile during both stair climbing and running.

See the Supplementary Video S1.

## 8 Experiment

In order to validate the proposed scheme on a physical platform, we tested the algorithm on the small humanoid OP3 by ROBOTIS. The same parameters adopted for the HRP-4 simulations were used, except for the sampling time *δ* = 0.025 s (used both by the MPC and the kinematic controller), $f_{z}^{\text{min}}=5$ N, the dimensions of the moving constraint region, that are set to *d*_*z*,*x*_ = *d*_*z*,*y*_ = 0.05 m, as well as those of the kinematically admissible region, that have been changed to *d*_*a*,*x*_ = 0.3 m, *d*_*a*,*y*_ = 0.05 m, with lateral displacement *ℓ* = 0.1 m.

The environment is composed of two ground patches, that is, the floor on which the robot is initially standing and slightly elevated ground. Along its path, the robot will encounter an overhanging obstacle, which has to be avoided by lowering the CoM. The footstep plan is generated with the following parameters: *α* = 0.4, $\bar{T}=0.6$ s, $\bar{L}=0.07$ m, *L*_max_ = 0.15 m. The reference velocity is chosen as 0.12 m/s. The candidate step length in the *x* direction is 0.07 m, while in the *y* direction the distance between the footsteps is 0.09 m. The reference CoM height is set to 0.22 m at the beginning of the experiment, lowered to overcome the obstacle, and then increased again.

The algorithm runs on the robot onboard computer (INTEL NUC with Intel Core i3-7100U 2 × 2.40 GHz processor and 8 GB 2133 MHz RAM) in real time. Each QP has a total of 28 decision variables (*T*_*c*_/*δ* = 0.7/0.025), while the number of constraints varies depending on which are active at a particular time (see Figure 8): QP-*z* always has exactly 28 constraints, while both QP-*x* and QP-*y* oscillate around 60.

**FIGURE 15:**
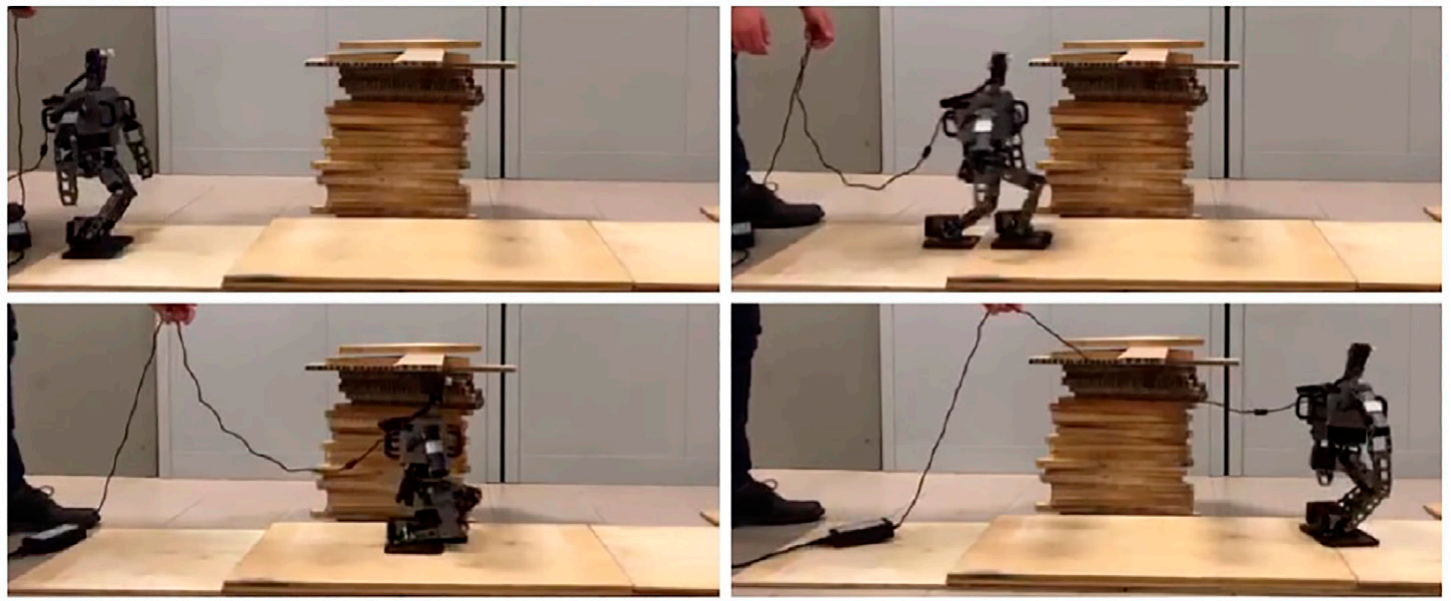
Snapshots from the experiment. OP3 walks forward and steps onto an elevated ground patch then lower its CoM to crouch in order to pass below an obstacle. The cable attached to the robot’s back is used for power supply only and does not sustain the robot while walking.

Snapshots of the experiment are reported in Figure 15. This experiment validates the proposed scheme on a small-sized humanoid, demonstrating its practical usability on a different platform with respect to the HRP-4 adopted for the dynamic simulations; see also the Supplementary Video S1.

## 9 Running Over Tilted Surfaces

The proposed method has been presented in the context of a world of stairs, where all contact surfaces are parallel. In this section, we show that it can be easily extended to the case of running over tilted surfaces. Although it would be possible to devise an extension for performing both walking and running motions, considering the case of running simplifies the problem. The reason is that, in a running gait, the robot is in contact with the tilted ground with a single foot at a time, and there is no double support phase. This is convenient because introducing double support over tilted surfaces would require adapting the definition of moving constraint to the case of nonparallel contact surfaces.

In Section 5.2 we considered friction by means of a constraint on the vertical GRF (9). Avoiding slipping on tilted surfaces requires higher friction because the tangential component of the GRF must also compensate for the tangential component of gravity. In order to account for this, it is sufficient to increase the minimum required vertical GRF by multiplying the right side of (9) by an appropriate coefficient which depends on the maximum slope of the ground. Since the environment is fully known, this can also be done on a per-footstep basis, by increasing the minimum GRF only when the foot is on a tilted surface, resulting in a less conservative GRF profile.

In order to maintain a formulation that is as close as possible to that presented in Section 5, we express the position of the CoM and ZMP in a frame that has the same orientation of the tilted patch (the *z* axis is the normal to the patch plane). In this frame, the vertical CoM dynamics assumes the expression:

$${\ddot{z}}_{c}=\frac{f_{z}^{\prime }}{m}-g_{z}^{\prime },$$ (25)

and the horizontal dynamics become:

$$\;{\ddot{x}}_{c}=\lambda \left(x_{c}-x_{z}\right)+g_{x}^{\prime },$$ (26)

$${\ddot{y}}_{c}=\lambda \left(y_{c}-y_{z}\right)+g_{y}^{\prime },$$ (27)

where $g_{x}^{\prime }$ , $g_{y}^{\prime }$ and $g_{z}^{\prime }$ correspond to the component of the gravity vector in the tilted plane frame. This choice provides a model that is structurally equivalent to (25–27), with the addition of the horizontal gravity terms acting as known disturbances.

Below, we modify the gait generation scheme described in Sections 5.2 and 5.3 by writing the prediction model based on (25–27) and performing an indirect compensation of the disturbance in the stability constraint (Smaldone et al., 2019).

In particular, the prediction model becomes:

$${\ddot{x}}_{c}=\left\{\begin{array}{cc} \lambda^{k+i}\left(x_{c}-x_{z}\right)+g_{x}^{\prime }\; & \text{for}\;t\in \left[t_{k+i},t_{k+i+1}\right)\;i=0,\ldots ,C-1\\ \lambda_{\text{LIP}}\left(x_{c}-x_{z}\right)+g_{x}^{\prime }\; & \text{for}\;t\geq t_{k+C}, \end{array}\right.$$ (28)

while the corresponding stability condition is given by the following result.

Proposition 3 Assume that |*x*_*z*_ (*t*′) − *x*_*z*_(*t*)| ≤ *a* + *b* (*t*′ − *t*), *∀t*′ ≥ *t*, for some *a*, *b* > 0, and that the current state $\left(x_{c}^{k},{\dot{x}}_{c}^{k}\right)$ satisfies

$$G\Phi \left(t_{k+C},t_{k}\right)\left(\begin{array}{c} x_{c}^{k}\\ {\dot{x}}_{c}^{k} \end{array}\right)+\int_{t_{k}}^{t_{k+C}}G\Phi \left(t_{k+C},\tau \right)B\left(\tau \right)x_{z}\left(\tau \right)d\tau =x_{u}^{k+C}-\int_{t_{k}}^{t_{k+C}}G\Phi \left(t_{k+C},\tau \right)Dg_{x}^{\prime }\left(\tau \right)d\tau ,$$ (29)

where the disturbance matrix is *D* = (0 1)^*T*^. Then, system (28) is internally stable, that is, *x*_*c*_ is bounded with respect to *x*_*z*_:

$$\exists M:\left|x_{c}\left(t\right)-x_{z}\left(t\right)\right|\leq M,\;t\geq t_{k}.$$

*Proof.* Following the same steps of the proof of Prop. 2 for system (28) and using (Smaldone et al., 2019)

$$x_{u}^{k}=\sqrt{\lambda_{\text{LIP}}}\int_{t_{k}}^{\infty }e^{-\sqrt{\lambda_{\text{LIP}}}\left(\tau -t_{k}\right)}x_{z}\left(\tau \right)d\tau -\frac{1}{\sqrt{\lambda_{\text{LIP}}}}\int_{t_{k}}^{\infty }e^{-\sqrt{\lambda_{\text{LIP}}}\left(\tau -t_{k}\right)}g_{x}^{\prime }\left(\tau \right)d\tau ,$$

we get (29).

Condition (29) can be written as a stability constraint as follows:

$$\begin{array}{c} G\overset{C-1}{\underset{i=0}{\sum }}\overset{C-1}{\underset{j=i}{\prod }}\Phi_{k+j}B_{k+i}x_{z}^{k+i}=\sqrt{\lambda_{\text{LIP}}}\int_{t_{k+C}}^{\infty }e^{-\sqrt{\lambda_{\text{LIP}}}\left(\tau -t_{k+C}\right)}{\tilde{x}}_{z}\left(\tau \right)d\tau -G\overset{C-1}{\underset{i=0}{\sum }}\overset{C-1}{\underset{j=i}{\prod }}\Phi_{k+j}Dg_{x}^{\prime ,k+i}-G\overset{C-1}{\underset{i=0}{\prod }}\Phi_{k+i}\left(\begin{array}{c} x_{c}^{k}\\ {\dot{x}}_{c}^{k} \end{array}\right), \end{array}$$ (30)

and used in the horizontal QP whenever required in order to step on tilted surfaces. Recall that the disturbance terms in (30) depend only on the tilting angle of the inclined planes and this information is assumed to be available from the footstep plan^9^.

### 9.1 Running Over Tilted Surfaces: Simulation

In this simulation the robot must run in an environment with two ramps (inclined by ± 8°) placed across the *x* path. A footstep plan is generated in response to the input velocity *v* = 0.35 m/s, which is switched to 1.1 m/s before the first ramp and then set back to the initial value after the second. We adopted the following parameters for the footstep planner: *α* = 0.4, $\bar{T}=0.7$ s, $\bar{L}=0.3$ m, *L*_max_ = 0.35 m.

**FIGURE 16:**
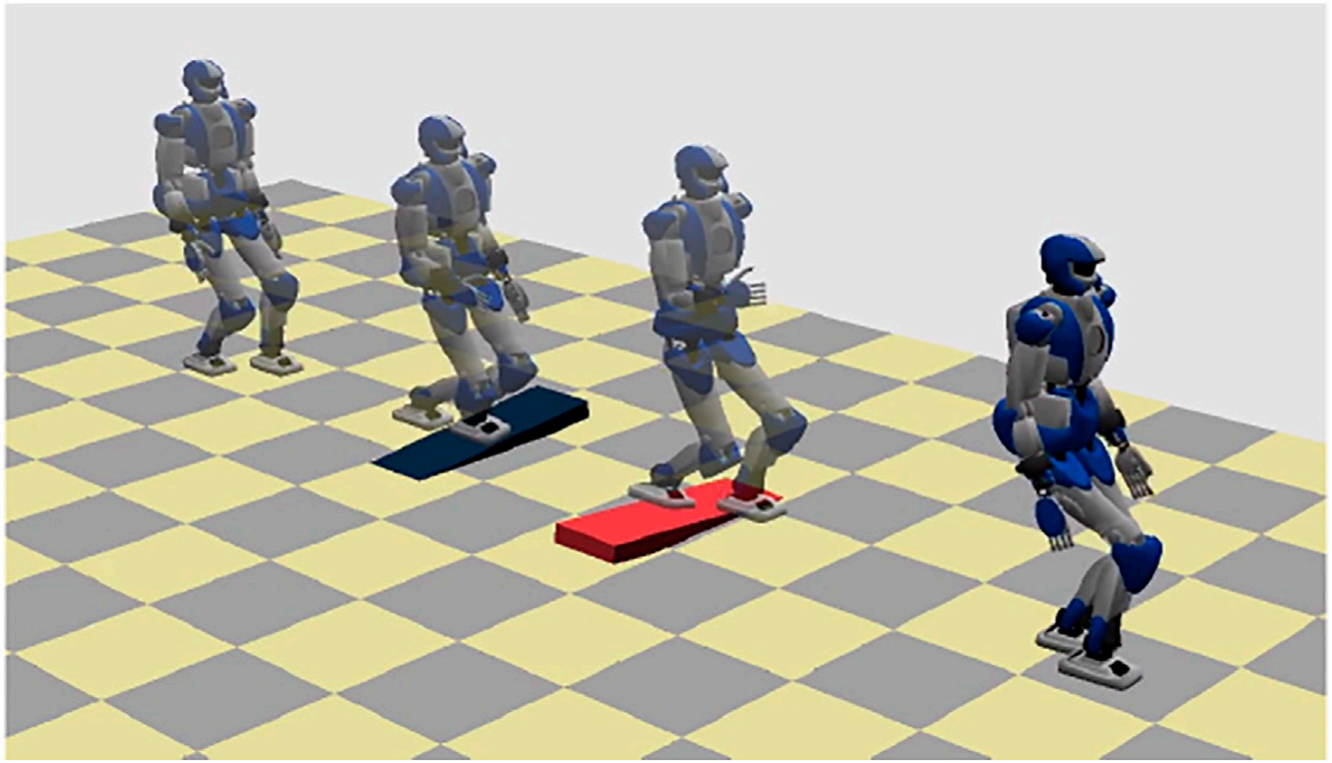
Running over tilted surfaces: stroboscopic motion.

**FIGURE 17:**
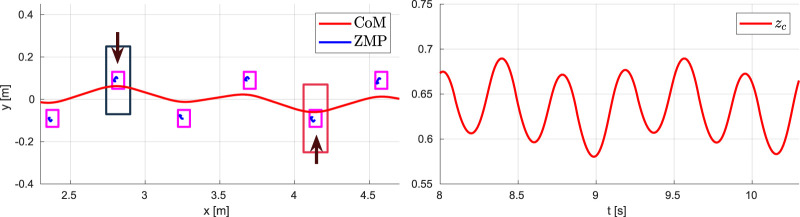
Running over tilted surfaces. CoM/ZMP trajectories (left) and CoM height (right). The plots only refer to the portion of the simulation before and after the two tilted surfaces (represented as rectangles in the CoM/ZMP plots). The black arrow represents the equivalent disturbance caused by the tangential component of the gravity when stepping on the tilted patch.

Results are shown in Figures 16, 17. The CoM/ZMP trajectories, representing five steps of the running gait, highlight the typical effect of the proposed form of indirect disturbance compensation, consisting of a CoM trajectory that leans against the effect of the perturbation introduced by the tilted surface.

## 10 Conclusion

We presented a gait generation algorithm that extends our IS-MPC to both the case of 3D walking and running gaits. The proposed scheme is articulated as a two-stage MPC, and features a new stability constraint for the VH-IP model, ensuring bounded CoM trajectories with respect to the ZMP. A simple planner which provides the required input for the scheme is provided, as well as an extension to the case of running over tilted surfaces. Dynamic simulation on HRP-4 and an experiment on the OP3 humanoid are presented in order to validate the framework.

The strengths of the presented method can be summarized as:

• the effect of the CoM height is fully accounted for in the balance condition, without the need to bound height variations or approximating the horizontal dynamics;
• the resulting scheme is completely formulated as a set of quadratic optimizations subject to linear constraints, which allows for a very efficient MPC implementation, as demonstrated by it running onboard on the OP3 humanoid robot;
• the stability constraint included in the formulation provides a guarantee of bounded CoM/ZMP trajectories;
• the generated trajectories are very natural and compatible with what is observed in human walking/running, as reported by studies in biomechanics.

Future work will be aimed at extending the framework in order to include features to achieve robustness, as well as the integration of the scheme with a more sophisticated footstep planner. Furthermore, the time-varying formulation of the stability constraint can be abstracted from the specific setting of variable height gait generation, and possibly applied to a different scenario modeled as a time-varying unstable system.

## Data Availability

The raw data supporting the conclusion of this article will be made available by the authors, without undue reservation.

## Footnotes

1 Other studies use the Centroidal Moment Pivot (CMP) point (Popovic et al., 2005) instead of the ZMP for gait generation and/or push recovery, for example, Griffin et al. (2017) and Shafiee-Ashtiani et al. (2017). The CMP purely encodes linear forces and thus does not require to assume the derivative of the centroidal angular momentum to be zero.

2 Throughout the article, points are represented by their coordinate vectors.

3 An alternative (Zamparelli et al., 2018) is to choose *z*_*z*_ as a control input in such a way that *λ* is constant. The approach adopted in this article is less constraining in terms of the CoM trajectories.

4 For compactness, we do not discuss in detail the module which provides the swing foot trajectory. It uses polynomial functions whose endpoints are adjusted in order to match the footstep positions, for example, see (Scianca et al., 2017).

5 Having two different horizons for planning and control is a common occurrence, as the window over which the planner operates is generally independent of the window over which the MPC optimizes. In fact, the first can range from a few steps up to the total duration of the locomotion task, while the second is usually much shorter in order to make the problem computationally tractable online. Furthermore, as shown in Scianca et al. (2020), having *T*_*c*_ < *T*_*p*_ is necessary to achieve recursive feasibility, as the information in the preview horizon is used to mitigate the effect of the limited control horizon on the stability of the generated trajectories.

6 The choice of rectangular regions simplifies the exposition, but the reasoning can be generalized to any convex shape.

7 This prevents continuous stair climbing and descending, resulting in a less fluid but more robust gait. Such choice is quite common for experimental humanoid platforms (Caron et al., 2019b).

8 A practical method to validate the result of our scheme on an actual robotic platform is to run a set of dynamic simulations. The generated motions can be evaluated based on the joint torques necessary to realize them, which can be directly obtained by querying the simulator. If the torques turn out to be excessive, one can try to modify some of the parameters in order to find a set that allows to produce a realizable motion (our results indicate that the duration of flight phases and step length are the most critical).

9 This information is in practice also used to generate a swing foot trajectory that is consistent with the slope of the terrain.
