# Supplementary material for: From Walking to Running: 3D Humanoid Gait Generation via MPC
Source: Front Robot AI. 2022 Aug 16;9:876613. doi: 10.3389/frobt.2022.876613 (PMC9446890; doi:10.3389/frobt.2022.876613)
Supplement: Supplementary file 1 [file Presentation1.pdf]

Appendix to From Walking to Running: 3D Humanoid Gait Generation via MPC

Filippo M. Smaldone, Nicola Scianca*, Leonardo Lanari and Giuseppe Oriolo

Dipartimento di Ingegneria Informatica, Automatica e Gestionale
Sapienza University of Rome, Italy

Correspondence*:
Nicola Scianca
scianca@diag.uniroma1.it

Proof of Proposition 1

The unstable subsystem dynamics (19), starting from the initial condition (20), gives the following state evolution

$$x_u(t) = \sqrt{\lambda_{\text{LIP}}} \int_t^{\infty} e^{-\sqrt{\lambda_{\text{LIP}}}(\tau - t)} x_z(\tau) d\tau.$$

By adding and subtracting $x_z(t)$ inside the integral we get

$$x_u(t) = \sqrt{\lambda_{\text{LIP}}} \int_t^{\infty} e^{-\sqrt{\lambda_{\text{LIP}}}(\tau - t)} (x_z(\tau) + x_z(t) - x_z(t)) d\tau,$$

after which we can separate and solve the integral only for $x_z(t)$ (which is independent of τ), and obtain

$$x_u(t) - x_z(t) = \sqrt{\lambda_{\text{LIP}}} \int_t^{\infty} e^{-\sqrt{\lambda_{\text{LIP}}}(\tau - t)} (x_z(\tau) - x_z(t)) d\tau.$$

The term inside the integral can be bounded using the hypothesis on the ZMP trajectory, i.e. $|x_z(t') - x_z(t)| \leq a + b(t' - t)$. After solving the integral, we get

$$|x_u(t) - x_z(t)| \leq \sqrt{\lambda_{\text{LIP}}} \left| \int_t^{\infty} e^{-\sqrt{\lambda_{\text{LIP}}}(\tau - t)} (x_z(\tau) - x_z(t)) d\tau \right| \leq a + \frac{b}{\sqrt{\lambda_{\text{LIP}}}} = M_u.$$

Note that this also implies, from (19), that $|\dot{x}_u| \leq \sqrt{\lambda_{\text{LIP}}} M_u$.

The dynamics of the CoM can be rewritten in terms of the evolution of x_u, giving

$$\dot{x}_c = -\sqrt{\lambda_{\text{LIP}}}(x_c - x_u),$$

which, by treating x_u as an input, evolves as

$$x_c(t) = x_c(t_0) e^{-\sqrt{\lambda_{\text{LIP}}}(t - t_0)} + \sqrt{\lambda_{\text{LIP}}} \int_{t_0}^{t} e^{-\sqrt{\lambda_{\text{LIP}}}(t - \tau)} x_u(\tau) d\tau,$$

where t_0 is the time at which the algorithm is initialized. Expanding the integral by parts and using the triangle inequality ($|\alpha + \beta| \leq |\alpha| + |\beta|$, $\forall \alpha, \beta \in \mathbb{R}$) allows to bound the difference between x_c and x_u as

$$|x_c(t) - x_u(t)| \leq \left| (x_c(t_0) - x_u(t_0)) \, e^{-\sqrt{\lambda_{\text{LIP}}}(t - t_0)} \right| + \left| \int_{t_0}^{t} e^{-\sqrt{\lambda_{\text{LIP}}}(t - \tau)} \dot{x}_u(t) d\tau \right| \leq S + M_u,$$

having used the bound on $\dot{x}_u$ and having defined $S = |x_c(t_0) - x_u(t_0)|$. Note that S can often be assumed to be zero, as the robot is usually not moving at t_0.

A bound on the CoM/ZMP displacement is obtained as follows,

$$|x_c(t) - x_z(t)| \leq |x_c(t) - x_u(t)| + |x_u(t) - x_z(t)| \leq S + 2M_u = M,$$

proving the thesis. ∎
